# Physical activity levels and associated socio-demographic factors in Bangladeshi adults: a cross-sectional study

**DOI:** 10.1186/s12889-016-4003-z

**Published:** 2017-01-11

**Authors:** Mohammad Moniruzzaman, M. S. A. Mansur Ahmed, Mohammad Mostafa Zaman

**Affiliations:** 1 Department of Community Medicine, Bangladesh University of Health Sciences (BUHS), Dhaka, Bangladesh; 2 Present Address: National Consultant for Injury and Disability Prevention, Noncommunicable Disease Unit, WHO Country Office for Bangladesh, United House (Ground to 3rd floor), 10 Gulshan Avenue, Gulshan-1, Dhaka, 1212 Bangladesh; 3 Division of Noncommunicable Diseases, Ekhlaspur Centre of Health (ECOH), Chandpur, Bangladesh

**Keywords:** Physical inactivity, Insufficient physical activity, Physical activity, Prevalence, Determinants, Correlate, Metabolic Equivalent Tasks (METs), GPAQ

## Abstract

**Background:**

Low level of physical activity (PA) has become an important public health problem even in low-income countries. The objectives of this study were to measure PA levels, determine the prevalence of low PA and identify socio-demographic factors associated with it in Bangladeshi adults.

**Methods:**

Data from 792 (urban, 395; rural, 397) Bangladeshi adults (25–64 years) were included in this population-based cross-sectional study conducted in 2011. Global Physical Activity Questionnaire version 2 (GPAQ-2) was used to measure PA. The metabolic equivalent task (MET) in minutes per week was calculated to determine total PA. Participants were categorized into low, moderate and high PA groups. Logistic regression was used to assess socio-demographic factors associated with low level of PA.

**Results:**

Median MET-minute of total PA per week was almost double in the rural area (1720) than the urban area (960). The overall prevalence of low PA was 50.3% (95% CI: 46.8–53.8), urban 59.5% (54.7–64.3) and rural 41.9% (37.0–46.8). Women in general were more inactive (women 63.1% [58.3–67.9], men 39.3% [34.6–44.0]). The main contributions to total PA were from work (urban 40.0%, rural 77.0%) and active commute (57.0%, 21.0%). Leisure-time PA represented a very small proportion (<3.0%). Multiple logistic regressions found a significant association of urban residence (OR = 2.2; 95% CI: 1.5–3.2), women (2.1; 1.4–3.9), oldest age group 55–64 years (15.6; 7.5–32.2) compared to youngest age group 25–34 years, graduation or further education (8.6; 4.1–17.7), and higher socio-economic class (2.4; 1.4–4.2) compared to poor with insufficient PA.

**Conclusions:**

This study identifies low PA in a rural and urban population in Bangladesh and that further large-scale population studies are warranted.

## Background

In recent years, physical activity (PA) has become an important public health issue in both high- and low-income countries [1, 2]. Indeed, regular PA is well known to be beneficial for health and well-being [3, 4]. There is strong evidence that it reduces rates of all-cause mortality and a number of noncommunicable diseases (NCDs) [5, 6]. Evidence suggests that risk reduction routinely occurs in adults when at least 150 minutes of moderate- to vigorous-intensity activity are ensured per week [7–14]. Physical inactivity, on the other hand, is indicated as a major risk factor for morbidity and mortality in adults [15, 16]. It has identified as an independent and fourth-leading risk factor for global mortality (6.0% of global deaths) [17]. Over the years global deaths due to physical inactivity have increased from 1.9 million in 2005 [18] to 3.2 million in 2008 [19] and 5.3 million in 2012 [5]. Different studies suggest that the prevalence of physical inactivity is rising in both high and low-income countries [1, 5, 19–23].

NCDs are increasing in Bangladesh [24]. This increase is concomitant to increasing sedentary lifestyle due to gradual mechanization of life in addition to the high prevalence of other major risk factors such as tobacco use [25] and salt intake [26]. Internationally comparable data on PA using standardized methods is still suboptimal in Bangladesh. Two studies [27, 28] reported the prevalence of physical inactivity 35.0% to 38.0% in Bangladeshi adults aged 25 years and older. However, earlier studies did not investigate the socio-demographic factors associated with insufficient PA, which is important information for planning of health-promoting interventions. Therefore, the objectives of this study were to measure PA levels and determine associated socio-demographic factors in Bangladeshi adults.

## Methods

### Study design and sampling

A population-based cross-sectional study was conducted among urban and rural Bangladeshi adults aged 25–64 years in 2011. A total of 806 individuals (equal numbers from the urban and rural area) were selected for interview by a two-stage systematic cluster sampling. Finally, data from 14 (1.7%) participants were identified as invalid during data cleaning, resulting in a final sample of 792 participants (urban 395, rural 397) for the current analysis. Data of 14 participants were removed if the value for at least one sub-domain (vigorous work, moderate work, transport, vigorous recreation, or moderate recreation activity) accounted more than 16 h; reported implausible values (eg. > 7 days in any days column); had inconsistent answers (e.g. 0 days but values > 0 in the corresponding time variables) as per GPAQ data cleaning guidelines [29].

#### Urban sampling

We randomly selected three mahallas—*the lowest urban geographic unit having identifiable boundaries*—out of total 10 mahallas from two purposively selected wards of Dhaka City Corporation, consisting of 382 holding numbers. Each household was considered as a cluster and who were available in that cluster were approached to participate in this study. Eligibility criteria included Bangladesh nationals aged 25–64 years who stayed in the household the night before the day of the first visit. Individuals who were mentally challenged (inability to communicate) or remained bed-ridden were excluded. Finally, a total of 96 holding numbers starting from holding number 1 were visited to get the targeted sample size (403).

#### Rural sampling

We purposively selected two villages of Tangail District—situated approximately 130 km northwest of the capital city, Dhaka. There were 467 households in these two villages. The same procedure was followed as in urban area for finding the eligible household in order to collect data. Finally, a total of 212 households were visited to get the targeted sample size (403).

### Data collection instrument

We used the Global Physical Activity Questionnaire version 2 (GPAQ-2) for measuring PA levels of this study [29]. It was developed by the WHO for PA surveillance in developing countries. The questionnaire consists of 16 questions covering moderate- and vigorous-intensity PA participation in three settings—PA at work, commuting (travel to and from places), and recreational activities as well as sedentary behavior. Vigorous-intensity activities were defined as “activities that require hard physical effort and cause large increases in breathing or heart rate”, and moderate-intensity activities were defined as “activities that require moderate physical effort and cause small increases in breathing or heart rate”. The participants were asked whether they engaged in these types of activities for at least 10 min continuously and, if so, for how many days (frequency) they performed these activities in a typical or usual week, and for how much time (intensity) they spent on a typical day etc. [29]. Question on sedentary behavior inquired only on time that they usually spent sitting or reclining on a typical day but did not include time spent sleeping [29].

For this study, we translated GPAQ-2 questionnaire into Bengali. Firstly, the questionnaire was translated from English to Bengali by two independent experts and then back translation was done from Bengali to English by another two independent experts. It was then finalized through a consensus meeting among these four experts. We applied both forward and backward methodologies during translation in order to ensure the appropriate meaning of each item was retained. No changes were made to the original contents; however, local examples of types and intensity of activities were used to suit the Bangladeshi context.

Field enumerators underwent a 2-day training before deployment. A list of moderate and vigorous activities for work and recreation domains with local examples were prepared and data collectors were adequately oriented with these activities including their operational definitions so that consistent findings could be obtained from both urban and rural settings. The GPAQ-2 analysis protocol was followed for data collection, processing, and analysis [29].

#### Conversion of PA data to estimated energy expenditure

We converted inquired data on PA (Total time spent on work, commuting and leisure-time activities of each intensity) to METs (Metabolic Equivalent Tasks), weighted by GPAQ-assigned MET energy expenditure ratios per kilogram per hour of 4 for moderate, and 8 for vigorous intensity activities. MET is the unit used to express the intensity of physical activities. Detailed methods of calculating MET have been described elsewhere [21, 27, 29].

#### Procedures for classifying PA levels

A person’s normal level of PA was classified into the low, moderate, and high level as defined by the GPAQ-2; the criteria of these levels have been described elsewhere [21, 27, 29].

We further categorized these three PA levels into **‘sufficiently active’** or **‘insufficiently active’** groups. The ‘sufficiently active’ group included participants who met the PA recommendation, therefore classified as being in the moderate or high level category (21, 29).

#### Socio-demographic factors

Information on the location of residence, sex, age, occupational status, educational levels, and socio-economic status were obtained in order to assess the association of these socio-demographic factors with insufficient PA. In regard to level the socio-economic status, participants were asked to allocate themselves to one of three categories; under which category (poor, middle class, and rich) they fall in.

### Data analysis

Median (interquartile range) of METs for sexes and urban-rural areas were obtained. The prevalence of PA levels and other categorical variables are reported as percentages with 95% confidence intervals (CIs). Finally, the binary logistic regression model was used to estimate relationships between physical inactivity and socio-demographic characteristics. Variables included in the model were the location of residence, sex, age groups, occupational status, educational level, and socio-economic status. Data were analyzed using SPSS version 16.0.

### Ethical consideration

Ethical clearance was obtained from the ethical review committee of Diabetic Association of Bangladesh (BADAS). International ethical guidelines for biomedical research involving human subjects were followed throughout the study [30]. Written (or thumb impression if unable to write) consent was obtained from all participants. Total 48 (women, 33; men 15) participants (about 6%) were unable to write.

## Results

Of total 792 (urban, 395; rural, 397) participants, 48.1% were women. The mean (standard deviation) age of the participants was 37.3 (10.4) years. One-fourth (23.4%) of the participants had no formal education and another one-third (35.2%) had completed any primary level education. Half of men (51.3%) were self-employed and one-third (37.5%) were salarymen both in public and nonpublic sectors. Of the women, 79.5% were homemakers. Further detail of socio-demographic information is given in Table 1.

**Table 1:** Socio-demographic background (results in %) of the study participants

Socio-demographic factors	Both sexes	Women	Men	*P* value
	(*n* = 792)	(*n* = 381)	(*n* = 411)	(Chi-Square)
Area of residence				
Urban	49.9	46.2	53.3	0.47
Rural	50.1	53.8	46.7	
Age groups (in years)				
25–34	44.6	48	41.4	0.00
35–44	28.5	32.3	25.1	
45–54	17.8	13.1	22.1	
55–64	9.1	6.6	11.4	
Occupational status				
Employed	26.6	15.0	37.5	0.00
Self-employed	27.1	1.0	51.3	
Student	3.5	2.1	4.9	
Housewife	38.3	79.5	0.0	
Unemployed	4.4	2.4	6.3	
Educational levels				
No formal schooling	23.4	28.9	18.2	0.00
Less than primary	8.6	9.2	8.0	
Primary completed	26.6	31.8	21.9	
Secondary completed	16.7	16.0	17.3	
Higher secondary completed	11.4	7.1	15.3	
Graduation degree and above	13.4	7.1	19.2	
Socio-economic status^a^				
Poor	32.3	34.6	30.2	0.40
Middle Class	51.6	49.9	53..3	
Rich	16.0	15.5	16.5	

^a^Self reported

### PA levels

#### Distribution of total PA MET-minute

The overall median MET-minute of total PA in a typical week was 1280. It was almost double in the rural area (1720) than the urban area (960). Men also reported double (1680) compared to women (800). These differences were statistically significant (*p* < 0.5).

#### Composition of total PA

**Fig. 1:**
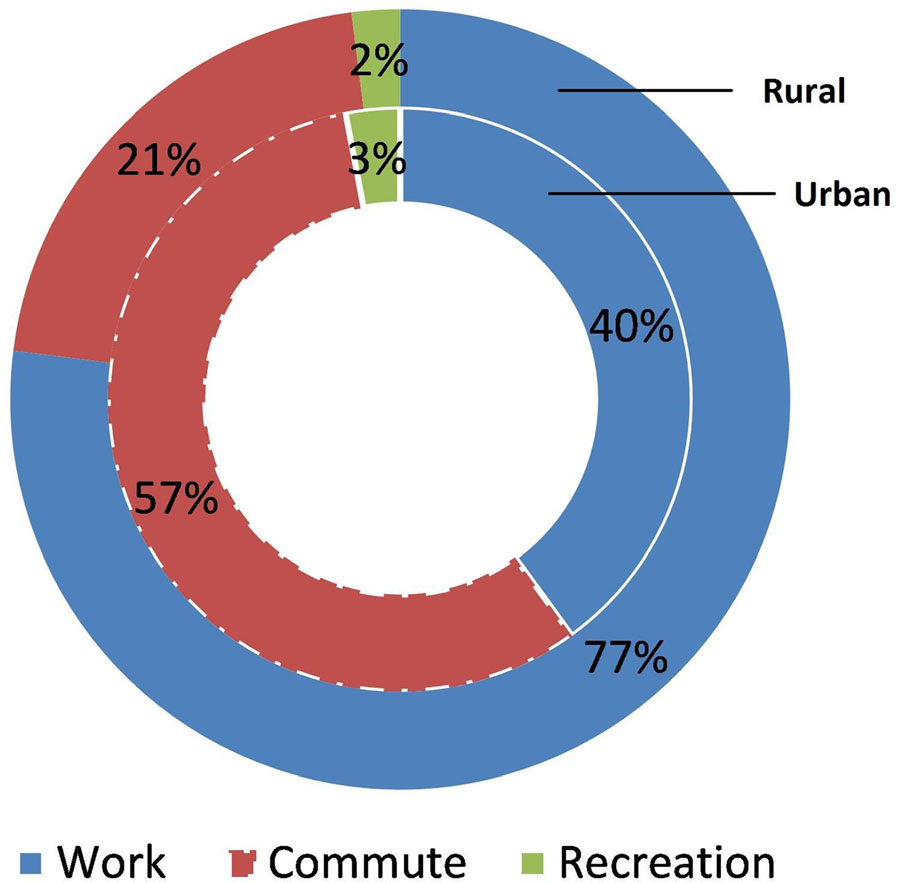
Composition of total physical activity in urban and rural areas

The compositions of total PA in both urban and rural areas were constituted mostly from work and commute domains. A little contribution was from leisure-time activity. Of total PA in urban, 40.0% was contributed by work-related activity followed by commuting (57.0%) and recreational activity (3.0%), whereas in rural, the composition was work-related activity (77.0%), commuting (21.0%) and recreational activity (2.0%) as shown in Fig. 1.

#### Prevalence of PA levels (low, moderate, and high)

According to the GPAQ-2 classification, the overall age-standardized prevalence of PA levels was low 50.3% (95% CI: 46.8–53.8), moderate 26.6% (23.5–29.7), and high 23.1% (20.6–26.0). The prevalence of low PA was significantly higher in urban areas (59.5%) than rural areas (41.9%). The prevalence of moderate and high level of PA in rural areas (31.8%, 26.3%) was comparatively higher than urban areas (21.3%, 19.2%) (Table 2).

**Table 2:** Prevalence of physical activity levels in urban and rural area,%

		Urban				Rural				Overall			
Sex	Age (years)	n	Low	Moderate	High	n	Low	Moderate	High	n	Low	Moderate	High
Men	25–34	110	31.8	31.8	36.4	60	26.7	25.0	48.3	170	30.0	29.4	40.6
	35–44	53	30.2	47.2	22.6	50	20.0	28.0	52.0	103	25.2	37.8	36.9
	45–54	33	57.6	18.2	24.2	58	32.8	24.1	43.1	91	41.8	22.0	36.3
	55–64	23	82.6	13.1	4.3	24	70.8	29.2	0.0	47	76.6	21.3	2.1
	25–64 (Crude), (95% CI)	229	40.6 (34.2–47.0)	31.5 (25.5–37.5)	27.9 (22.1–33.7)	192	32.3 (25.7–38.9)	26.0 (19.8–32.2)	41.7 (34.7–48.7)	411	36.7 (32.0–41.4)	29.0 (24.6–33.4)	34.3 (29.7–38.9)
	25–64 (age standardized)^a^, (95% CI)	229	45.9 (39.4–52.4)	29.8 (23.9–35.7)	24.3 (18.7–29.9)	192	33.7 (27.0–40.4)	26.3 (20.1–32.5)	40.0 (33.1–46.9)	411	39.3 (34.6–44.0)	28.6 24.2–33.0)	32.1 (27.6–36.6)
Women	25–34	76	60.5	13.2	26.3	107	46.7	39.3	14.0	183	52.5	28.4	19.1
	35–44	65	72.3	12.3	15.4	58	27.6	55.2	17.2	123	51.2	32.5	16.3
	45–54	26	92.3	7.7	0.0	24	54.2	25.0	20.8	50	74.0	16.0	10.0
	55–64	9	77.8	22.2	0.0	16	93.8	6.2	0.0	25	88.0	12.0	0.0
	25–64 (Crude), (95% CI)	176	70.5 (63.8–77.2)	12.5 (7.6–17.4)	17.0 (11.5–22.5)	205	45.9 (39.1–52.2)	39.5 (32.8–46.2)	14.6 (9.8–19.4)	381	57.3 (52.3–63.3)	27.0 (22.5–31.5)	15.7 (12.0–19.4)
	25–64 (age standardized)^a^, (95% CI)	176	74.1 (67.6–80.6)	13.2 (8.2–18.2)	12.7 (7.8–17.6)	205	51.0 (44.2–52.7)	34.8 (28.3–41.3)	14.2 (9.4–19.0)	381	63.1 (58.3–67.9)	23.9 (19.6–28.2)	13.0 (9.6–16.4)
Both	25–34	186	43.5	24.2	32.3	167	39.5	34.2	26.3	353	41.6	28.9	29.5
	35–44	118	53.4	28.0	18.6	108	24.1	42.6	33.3	226	39.4	35.0	25.6
	45–54	59	72.8	13.6	13.6	82	39.0	24.4	36.6	141	53.2	19.8	27.0
	55–64	32	81.3	15.6	3.1	40	80.0	20.0	0.0	72	80.5	18.1	1.4
	25–64 (Crude), (95% CI)	395	53.9 (49.0–58.8)	23.1 (18.9–27.3)	23.0 (18.8–27.2)	397	39.3 (34.5–44.1)	33.0 (28.4–37.6)	27.7 (23.3–32.1)	792	46.6 (43.1–50.1)	28.0 (24.9–31.1)	25.4 (22.4–28.4)
	25–64 (age standardized)^a^, (95% CI)	395	59.5 (54.7–64.3)	21.3 (17.3–25.3)	19.2 (15.3–23.1)	397	41.9 (37.0–46.8)	31.8 (27.2–36.4)	26.3 (22.0–30.6)	792	50.3 (46.8–53.8)	26.6 (23.5–29.7)	23.1 (20.2–26.0)

^a^Standardized to the age distribution of the new WHO world standard population (2000–2025)

### Socio-demographic factors associated with low PA level

We also assessed whether PA associated with socio-demographic factors such as age, sex, occupation, education, and socio-economic status (Table 3). Overall urban dwellers were positively associated (OR = 2.2, [95% CI: 1.5–3.2]) with insufficient PA compared to those residing in rural areas. Generally, women and housewives, in particular, were 2.1 and 3.8 times more likely than men, and other occupations respectively to have an insufficient PA. Although OR for having insufficient PA increased across the age groups, the oldest age group reported greatest insufficient PA compared to the youngest age group, especially in urban areas (30.8, 8.4–113.2). Compared with those having no formal education, insufficient PA was 8.6 times higher in individuals who completed graduation or more. Overall, individuals self-allocated to the higher socio-economic class category were 2.4 times more likely than those self-allocated to the poor category to report insufficient PA as shown in Table 3.

**Table 3:** Odds ratio (95% confidence interval) of socio-demographic factors for insufficient physical activity

Socio-demographic factors	Both areas	Urban	Rural
	(*n* = 792)	(*n* = 395)	(*n* = 397)
Sex			
Men	Ref.	Ref.	Ref.
Women	2.1 (1.4–3.9)	3.6 (1.5–8.6)	1.4 (0.4–5.3)
Age groups (in years)			
25–34	Ref.	Ref.	Ref.
35–44	1.1 (0.7–1.6)	1.9 (1.0–3.6)	0.5 (0.3–1.0)
45–54	3.4 (2.0–5.6)	9.4 (3.5–25.2)	1.5 (0.8–2.8)
55–64	15.6 (7.5–32.2)	30.8 (8.4–113.2)	10.6 (4.2–26.5)
Occupational status			
Employed	Ref.	Ref.	Ref.
Self-employed	1.1 (0.6–1.9)	0.4 (0.2–1.0)	0.9 (0.3–2.7)
Student	1.4 (0.5–3.3)	0.6 (0.2–2.2)	2.3 (0.5–10.8)
Housewife	3.8 (2.0–7.2)	7.8 (3.2–18.7)	1.9 (0.4–8.8)
Unemployed	1.7 (0.7–4.1)	3.2 (0.9–11.6)	0.7 (0.2–3.5)
Educational levels			
No formal schooling	Ref.	Ref.	Ref.
Less than primary	1.2 (0.6–2.3)	2.2 (0.6–7.7)	0.8 (0.3–1.9)
Primary completed	2.4 (0.8–3.8)	3.4 (0.9–9.4)	2.0 (0.8–3.6)
Secondary completed	1.2 (0.7–2.1)	2.7 (0.9–8.0)	0.7 (0.3–1.4)
Higher secondary completed	4.5 (2.3–9.0)	17.0 (4.8–60.2)	1.6 (0.6–4.3)
Graduation degree and above	8.6 (4.1–17.7)	25.8 (7.7–86.4)	5.2 (1.3–20.6)
Socio-economic status^a^			
Poor	Ref.	Ref.	Ref.
Middle Class	1.4 (0.9–2.0)	2.1 (1.1–3.8)	0.9 (0.5–1.5)
Rich	2.4 (1.4–4.2)	6.7 (2.2–20.0)	1.3 (0.7–2.7)

^a^Self reported

## Discussion

The paper reports the prevalence of PA and socio-demographic factors associated with low level of PA among adults living in urban versus rural settings in Bangladesh using GPAQ-2. Irrespective of age and gender, half (50.3%) of the adult population showed a ‘low level’ of PA. A few national studies done using GPAQ on adults with similar age groups showed the prevalence of low PA of 34.5% [27], 38.0% [28] and 49.2% [31] while two other studies reported low PA which is not directly comparable because of using different approaches of leveling low PA (35.2%) [32] and/or unable to provide point estimate [33]. Results of these studies showed a wide variability with our estimate, which is a not uncommon phenomenon. A review demonstrated that PA estimates vary markedly even within a single country using different surveys in similar time periods [34]. Although our estimate of low PA is much higher than the global estimate (31.0%) of adults aged 15 years and above [35] it is similar to a recent prevalence estimate of our neighboring India (54.4%), whom population characteristics are similar to Bangladeshi population [36]. Data of Behavioral Risk Factor Surveillance System showed that half of the U.S. adults (49.4%) were physically inactive which is also similar to our current estimate [37]. Variability of low physical activity level between different regions of the world, among different countries and within the country is well documented [22, 34, 38–40].

This study also confirmed that urban adults are more physically inactive (or insufficiently active) than that in rural adults. It means that these people are not meeting the minimum recommendation of at least 30 minutes of moderate-intensity PA or walking for 5 or more days per week. This difference of physical inactivity between people living in the urban and rural area might be explained by poorly planned urbanization including mechanization of life, lack of play grounds, parks, walkable footpaths; unsafe roads for bicycles etc. [25]. The existence of mechanized work appliance, sedentary life-style and less engagement in vigorous-intensity activities like agricultural work in urban area might also have limited overall participation in PA.

In general, results of this study confirmed that urban dwellers, women, older age group, people with higher level of education, and higher socio-economic class are more likely to report insufficient PA. These observations are similar to the results observed in many low and middle incomes [1, 20–22, 25, 40, 41]. However, very few countries show dissimilarities in gender and age, for example, Croatia, Hungary, Slovakia, Kazakhstan, Ukraine, Argentina, Portugal and Saudi Arabia where men are more likely to report the low level of PA compared to women [20, 41]. Likewise, though there was a general decline across age groups, the rate of PA remained high in the older age group for some countries like New Zealand, China and Hong Kong [20, 41]. These differences can be explained by their unique lifestyle and workforce pattern.

In this study, in general, men reported spending more time in work and transport-related physical activities compared to women in both urban and rural areas. The results of this study also showed that physical activities at work and commute domains are the main contributors to total PA among the study population. In addition, physical activities undertaken as part of recreational or leisure-time activity contributed very little (around 3.0%) to the total PA in this population. These results are in line with many low and middle-income countries where work and transport-related activities are the prime contributors to overall PA compared to leisure time activities [21, 22, 38, 42]. However, in high-income countries (such as Australia, Canada, New Zealand, USA), leisure-time PA is a major component of total PA undertaken by adults [20, 43, 44].

This difference can be explained by a higher availability and accessibility to sports or recreational facilities and a history of long-term promotion of exercise in high-income countries. Moreover, PA through sports in leisure-time is a concept which is already well suited in high-income countries but not an established concept in many low-income countries like Bangladesh. This suggests that promoting leisure-time PA can be a strategy to increase overall PA at the population level in our country. Considering the growing burden of NCDs in our country, this potential risk factor that shows the higher prevalence in this study must need to address at national, community and individual level for combating this growing epidemic and improving the overall health of the population.

Certain factors might influence the findings of the current study like the subjective judgment of vigorous and moderate PA might differ by the location, the level of education and gender. Generally, people, especially in the rural setting, do not habituate to measure the time they spend on certain activities in hours and minutes. This phenomenon is also true for certain urban people who are engaged in the informal sector. Therefore, people might have under or over-reporting their physical activities that cannot be over ruled with certainty. Potential bias related to the categorization of socio-economic status cannot be over ruled because it was completely subjective. However, efforts were given to overcome these challenges by preparing exhaustive check list and adequate training of data collectors. Age-specific prevalence of PA should be interpreted cautiously because of inadequate sample size for age groups. Finally, the cross-sectional nature of the study design itself is one of the limitations in terms of identifying the associated factors. It is noteworthy here that our results on the basis of education could be considered as a proxy of socio-economic status.

Limitations aside, this study provides a valuable snapshot of domain specific (work, commute, and recreation) PA patterns for urban and rural adults of Bangladesh which will provide valuable information for public health intervention planning to promote PA at the population level to combat the growing epidemic NCDs in Bangladesh.

## Conclusion

Insufficient PA is highly prevalent among Bangladeshi adult population. Interventions targeting women, oldest age group, people with higher education, and higher socio-economic class especially in the urban area are warranted. The results of this study will focus the necessity of primary prevention of NCDs through PA intervention at the population level and will provide the baseline information about the PA levels of adult population in Bangladesh which will help the policy-makers at the national level to develop the national guidelines for PA to promote overall PA level.

## Abbreviations

CI: Confidence interval
GPAQ-2: Global Physical Activity Questionnaire version 2
MET: Metabolic Equivalent Task
NCDs: Noncommunicable diseases
PA: Physical activity
SPSS: Statistical Package for the Social Sciences
WHO: World Health Organization
